# Chemotherapy near the end of life: a retrospective single-centre analysis of patients’ charts

**DOI:** 10.1186/1472-684X-13-26

**Published:** 2014-05-22

**Authors:** Hanny Adam, Sonja Hug, Georg Bosshard

**Affiliations:** 1 Department of Oncology, City Hospital Waid, Tiechestrasse 99, CH-8037 Zurich, Switzerland; 2 Institute for Social Work and Health, University of Applied Sciences and Arts, Northwestern Switzerland FHNW, CH-4600 Olten, Switzerland; 3 Geriatrics Clinic, Zurich University Hospital, and Center for Age and Mobility, University of Zurich and City Hospital Waid, Rämistrasse 100, CH-8091 Zurich, Switzerland

## Abstract

**Background:**

Chemotherapy near the end of life is an issue frequently discussed nowadays. The concern is that chemotherapy could cause more harm than good in a palliative situation; this is even truer as the patient nears death. The objective of our study is to evaluate the aggressiveness of patient care near the end of life by determining how many cancer patients receive chemotherapy during their final weeks.

**Methods:**

In a retrospective analysis of patient charts, we investigated whether cancer patients had been treated with chemotherapy during the last four or two weeks of life. If they had, we looked at whether treatment was ongoing or newly initiated.

**Results:**

Out of the 119 cancer patients who died in our hospital over two years, 14 (11.7%) received chemotherapy during the last four weeks of life, nine of whom (7.6%) in the last two weeks of life. Treatment had been ongoing in six (5%) and newly initiated for eight (6.7%) within four weeks of death. Corresponding figures for the last two weeks of life were seven patients (5.9%) who continued previously prescribed treatment and two (1.7%) who were started on chemotherapy. Patients given chemotherapy during the last four weeks of life were significantly younger than those who were not (p = 0.003).

**Conclusions:**

Cancer patient care in our hospital is not considered overly aggressive as only 7.6% of these patients receive chemotherapy within the last two weeks of life. To determine how aggressive care near the end of life really is, however, we suggest evaluating newly started chemotherapy alongside ongoing treatment. As the line between the effects (beneficience) and side effects (nonmaleficience) of chemotherapy is often very narrow, doctors and patients have to work together to find the best way of treading this fine line.

## Background

Chemotherapy near the end of life is an issue frequently discussed nowadays. Both, patients and doctors are concerned that chemotherapy could cause more harm than good in a palliative situation. This is even truer as the patient nears death, when the main aim of treatment is usually palliation and not prolonging life.

Earle et al. report that the treatment of cancer patients near the end of life is becoming more and more aggressive [1]. According to the Health Service Research Committee of the American Society of Clinical Oncology (ASCO), treatment can be recommended if it improves the quality of life in patients with metastatic cancer even though it does not improve survival [2].

Clinical trials have shown that chemotherapy may palliate symptoms with a resultant improvement in quality of life. However, giving palliative chemotherapy near the end of life, is a balancing act between clinical benefit and potential harm in terms of side effects. Appropriately timed discontinuation of chemotherapy is essential for the patient’s quality of life. The ASCO Quality Oncology Practice Initiative (QOPI) included “Chemotherapy administered within the last two weeks of life (lower score-better)” as a benchmark for improving clinical practice [3].

Questions of particular interest are:

– What percentage of patients can receive chemotherapy near the end of life in such a way that care is not overly aggressive – and how long before death should chemotherapy be stopped?

– What constitutes a good treatment decision and who is involved in the decision- making process?

When ascertaining the number of patients receiving chemotherapy near the end of life, several authors have focused on the four weeks before death. In our study, we determined not only how many patients had been given chemotherapy within the last four weeks of life, but also how many had been treated within the last two weeks, as indicated by QOPI [3]. We mad**e** a distinction between ongoing treatment and newly initiated chemotherapy.

## Methods

Our hospital is a midsize public hospital in a city in Switzerland; cancer patients are cared for and treated as outpatients or inpatients by a team of oncologists. Using computerised medical records and manual searches in charts, we included all patients whose main diagnosis was cancer and who died in our hospital in 2006 or 2007. Our rationale for selecting patients with a main diagnosis of cancer was that, in theory, only these patients would have had an indication for chemotherapy. We concentrated on chemotherapy and did not analyse other treatment, such as radiotherapy or surgery.

Data gathered for our retrospective analysis included the following:

type of cancer

age of patients

sex of patients

chemotherapy during the last four weeks of life

– ongoing chemotherapy

– start of new chemotherapy

chemotherapy during the last two weeks of life

ongoing chemotherapy

start of new chemotherapy

All data were anonymised.

To calculate a possible correlation between the age or sex of a patient and the probability to be given chemotherapy near the end of life we used the Fisher’s exact test.

The Ethics Committee of the Canton of Zurich, Switzerland, approved this project.

We compared our results with those found in the literature, in order to discuss them from an ethical point of view in the light of other publications. At the same time, we reviewed the decision-making process.

## Results

The analysis took data from the clinical charts of 119 patients who died of cancer in our hospital during 2006 or 2007: 62 in 2006 and 57 in 2007.

### Patients’ characteristics

The median age of all 119 patients was 75 (range 48–94) years; 48 (40%) of all patients were women, 71 (60%) were men. The most frequent diagnosis in our patients was gastrointestinal cancer (44/119 patients; 37%), followed by lung cancer (36/119; 30%). Figures for gynaecological cancers (12/119; 10%) and urogenital cancers (7/119 patients; 6%) were lower in our patient population than would generally be expected, as our hospital has no gynaecology or urology department. Nine (8%) patients were suffering from lymphoma, leukaemia or myeloma, and 11 (9%) patients from other malignancies (Table 1).

**Table 1:** Patients‘ characteristics (N = 119)

**Typ of cancer**	**Numbers**	**%**
Gastrointestinal	44	37
Lung	36	30
Gynaecological	12	10
Lymphoma/leukaemia/myeloma	9	8
Urogenital	7	6
Others	11	9
**Gender**	**Numbers**	**%**
Female	48	40
Male	71	60
**Ethnicity**	**Numbers**	**%**
Caucasian	119	100
Other	0	0
**Age**	**Range**	**Median**
Years	48-94	75

### Chemotherapy near the end of life

During the last four weeks of life (day −28 until death) 14 patients (11.7%) were treated with chemotherapy. Six patients (5%) continued with their previously prescribed treatment and eight patients (6.7%) were started on chemotherapy.

During the last two weeks of life (day −14 until death) nine patients (7.6%) were given chemotherapy. Seven patients (5.9%) were already on chemotherapy, while two patients (1.7%) were started on chemotherapy during this time (Table 2).

**Table 2:** Chemotherapy at the end of life (last four and last two weeks)

**N = 119**	**Last 4 weeks**	**Last 2 weeks**
**New chemotherapy**	8 (6.7%)	2 (1.7%)
**Ongoing chemotherapy**	6 (5%)	7 (5.9%)
**Total**	14 (11.7%)	9 (7.6%)

**Figure 1:**
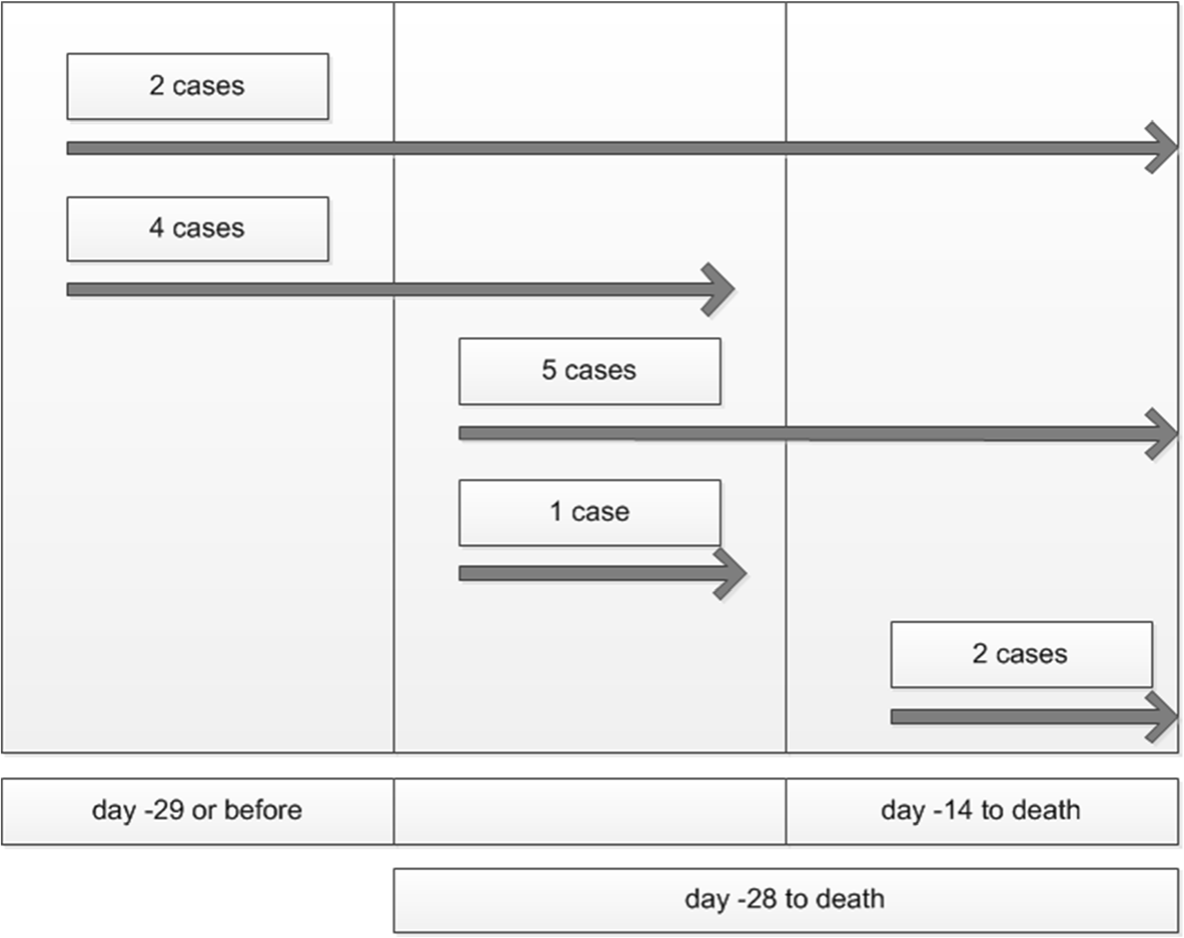
Treatment sequence (continued, started, stopped) during the last weeks of life.

Figure 1 shows the treatment sequence (continued, started, stopped) during the last weeks of life.

All chemotherapy was intended for the palliative care of patients whose cancer was considered incurable, with the main aims of relieving symptoms and improving the quality of life.

### Characteristics of patients receiving chemotherapy near the end of life

Among the 14 patients who received chemotherapy during the last four weeks of life, 11 (79%) were younger than 75 ( = median age); six (43%) were women.

We carried out a statistical analysis to examine possible associations between the patient’s age or sex and the fact that they were given chemotherapy during the last four weeks of life. Fisher’s exact test gave a highly significant p-value of 0.003 with respect of age. This means that the patients in our cohort given chemotherapy near the end of their lives (last four weeks) were significantly younger than those not receiving any chemotherapy. However, the patient’s sex had no effect on whether or not they were given chemotherapy near the end of life (p = 1.0) (Table 3).

**Table 3:** Age and sex of patients given or not receiving chemotherapy during the last four weeks of life

		**Chemotherapy during the last four weeks of life**		**p value Fisher’s exact test**
		**Yes (14 = 100%)**	**No (105 = 100%)**	
**Age**	**< 75 y**	**11 (79%)**	**37 (35%)**	**0.003**
	**≥ 75 y**	**3 (21%)**	**68 (65%)**	
**Sex**	**Male**	**8 (57%)**	**63 (60%)**	**1.0**
	**Female**	**6 (43%)**	**42 (40%)**	

Among the 14 patients given chemotherapy near the end of life, primary cancer sites were gastrointestinal in four, lung in three, myeloma or leukaemia in three, urogenital in two, gynaecological in one, and other in one.

### Comparison with published results on chemotherapy of cancer patients at the end of life

Out of the five published studies, three analysed the situation concerning chemotherapy during the last four weeks of life and gave figures of between 7.3% and 18.8% for all cancer patients (our result: 11.7%). Four papers also reported on chemotherapy given during the last two weeks of life, with percentages ranging from 4.2% to 11.6% (our result: 7.6%) (Table 4).

**Table 4:** Summary of reports in the literature concerning chemotherapy near the end of life compared with our own results

**Author**	**Näppä**	**Earle**	**Kao**	**Braga**	**Barbera**	**Our results**
**Country**	Sweden	USA Canada	Australia	Portugal	Canada	Switzerland
**No of patients**	1200	215′848	747	639	21323	119
**Period analysed (years)**	1	10	2	1	1	2
**Median age (years)**	65	≥ 65	67	61	72	75
**Sex f/m (%)**	44/56	NA	44/56	61/39	47/53	40/60
**Chemotherapy in last 4 weeks (%)**	7.3	NA	9.8	18.8	NA	11.7
				**4.7 *new***		**6.7 *new***
**Chemotherapy in last 2 weeks (%)**	NA	9.7-11.6	4.3	10.7	4.2	7.6
						**1.7 *new***

Näppä et al. [5], Kao et al. [6], Braga et al. [4], Barbera [7], Earle et al. [8].

Braga et al. [4] determined that 4.7% of all cancer patients had been started on chemotherapy within four weeks of death (our result: 6.7%). None of the authors investigated how many patients had received chemotherapy beginning during the last two weeks of life (our result: 1.7%).

## Discussion

This is the first study from Switzerland on the aggressiveness of cancer care in the last four/two weeks of life.

In a retrospective analysis of 119 clinical charts we determined the number of cancer patients in our Zurich municipal hospital who received chemotherapy near the end of life.

During the last four weeks (28 days) of life, 14 patients (11.7%) were given chemotherapy: ongoing treatment in six patients (5%) and newly initiated chemotherapy in eight patients (6.7%).

During the last two weeks (14 days) of life, nine patients (7.6%) received chemotherapy: seven (5.9%) continued with previously prescribed treatment, while two (1.7%) were started on chemotherapy.

The number of patients analysed in this study is relatively small (119), and the study sample is just from one hospital site. This means that the generalizability of the results of this study is limited. But still the results are important, as worldwide there is only a very limited number of studies on this issue.

A literature search revealed five studies presenting analyses comparable to our own. However, none of them made a distinction between ongoing and newly initiated chemotherapy, as we did.

Three of these five studies found in the literature reported the number of cancer patients on chemotherapy during the last four weeks of life [4-6], ranging from 7.3% to 18.8%. Only one author determined the cases (4.7%) in which chemotherapy had been started during this period [4].

Four of the five studies also reported on chemotherapy during the last two weeks of life [4,6-8], with figures ranging from 4.2% to 11.6% of patients, but none of them determined how often chemotherapy was actually started within two weeks of death.

Earle et al. generated and evaluated quality indicators for end-of-life cancer care, through a combination of literature reviews, focus groups, and assessment by an expert panel [9]. One of their three major concepts of poor quality end-of-life care is administering chemotherapy very close to death.

The same working group later published results of benchmarking assessments [10], concluding that “the analysis of SEER-Medicare claims suggests that health care systems not providing overly aggressive care would be ones in which less than 10% of patients receive chemotherapy in the last 14 days of life”. The findings of Kao et al. [6] with 4.3% and Barbera with 4.2% meet this criterion [7], as do ours with 7.6%. Earle et al. [8] describe an increasing number of patients receiving chemotherapy during the last two weeks of life from 9.7% in 1993 to 11.6% in 1999.

In our opinion, however, there is an important difference between ongoing and new chemotherapy. The reason for starting chemotherapy is always to treat cancer; it is always an active procedure. On the contrary, the reasons for not stopping chemotherapy can vary. For one thing patients may die from causes other than cancer, sometimes unexpectedly. On these grounds, starting new chemotherapy near the end of life is a more aggressive approach than simply not discontinuing ongoing treatment.

To determine how aggressive end-of-life care for cancer patients really is, we suggest that newly initiated chemotherapy should be evaluated separately from ongoing treatment.

And last but not least, the final weeks of life can be analysed only in retrospect. At the start of treatment, as well as during the treatment process, it is not always easy to estimate the lifetime remaining.

How does the decision-making process concerning chemotherapy near the end of life in fact evolve? Who decides about treatment in palliative cancer care, especially when the patient is near death? What are the prerequisites for considering chemotherapy near the end of life?

For doctors, the first prerequisite for chemotherapy is, of course, that we are dealing with a tumour that is sensitive to cytostatic treatment. The second one is that we can expect to see a reasonably rapid response in advanced stages of the disease. And we always have to take the patient’s general condition and comorbidities into account. For both doctors and patients, reports mention younger patient age as a predictor of the likely use of chemotherapy [4,11]. In our analysis, the patients who were treated with chemotherapy within the last four or two weeks of life were significantly younger than those who were not (p = 0.003).

The basics of good treatment decisions are the patient’s wishes and the doctor’s recommendations; functional communication between doctor and patient is essential. We have to be aware that communication is more than just delivering information [12]; it aims primarily to establish a relationship. It is important for patients to obtain transparent and useful information and they need guidance in understanding the issues involved. On the other hand, the patient’s individual wishes, preferences and moral concepts have to be respected. Both patient and doctor have to participate in this shared decision-making process [13]. In their own ways, both are experts; doctors know more about the theoretical context of a disease, but patients know how they feel actually having it. In a shared decision making-process, patient and doctor act as partners.

The line between effects and side effects of chemotherapy is often very narrow. In making treatment decisions in palliative cancer care, and especially near the end of life, we always have to bear the patient’s quality of life in mind. Patients have to tell us what quality of life means for them, as only they can know.

In accordance with bioethical principles, as published by Beauchamp and Childress [14], we do, of course, always keep beneficience in mind when we think about chemotherapy. We want patients to benefit from treatment. In palliative situations, however, the question is how much can we accept in the way of side effects when our aim is to help the patient? Treading the fine line between beneficience and nonmaleficience, between the effects of chemotherapy and its side effects, is a delicate balancing act. Only by consciously and responsibly discussing the matter with patients, and by respecting their wishes and their autonomy, can this balance be achieved.

## Conclusions

Cancer patient care in our hospital is not considered overly aggressive as only 7.6% of these patients receive chemotherapy within the last two weeks of life. To determine how aggressive care near the end of life really is, however, we suggest evaluating newly started chemotherapy alongside ongoing treatment. As the line between the effects (beneficience) and side effects (nonmaleficience) of chemotherapy is often very narrow, doctors and patients have to work together to find the best way of treading this fine line.

## Competing interests

There are no financial or non financial interests to declare in relation to this manuscript.

## Authors’ contributions

HA drafted the study design, conducted the data collection, took an active part in the analysis of data, as well as in the draft of the paper, the review and final approval. SH has been involved in the study design and the review and final approval of the paper. GB has contributed to the analysis of data, the draft of the paper, conducted the statistical analysis and participated in the review and final approval of the paper.

## Pre-publication history

The pre-publication history for this paper can be accessed here:

http://www.biomedcentral.com/1472-684X/13/26/prepub
